# Average relative flow of single-wing labyrinth drip irrigation tape based on projection pursuit regression

**DOI:** 10.1038/s41598-022-12638-y

**Published:** 2022-05-20

**Authors:** Hongfei Tao, Juanqin Tao, Qiao Li, Mahemujiang Aihemaiti, Youwei Jiang, Wenxin Yang, Jianqun Wei

**Affiliations:** 1 College of Hydraulic and Civil Engineering, Xinjiang Agricultural University, Urumqi, 830052 China; 2 Xinjiang Key Laboratory of Hydraulic Engineering Security and Water Disasters Prevention, Urumqi, 830052 China

**Keywords:** Fluid dynamics, Statistics

## Abstract

The hydraulic performance of single-wing labyrinth drip irrigation tapes under the coupling effect of water and fertilizer affects the operating efficiency of the entire drip irrigation system. In this study, three types of single-wing labyrinth drip irrigation tapes were studied. We evaluated the average relative flow of each type and conducted indoor uniform orthogonal tests of three factors, namely, fertilizer concentration, sediment content, and operating pressure. The results showed that the order of the factors affecting the average relative flow of single-wing labyrinth drip irrigation tape was sediment content > fertilizer concentration > operating pressure. The projection pursuit regression (PPR) models of the average relative flow of three types of single-wing labyrinth drip irrigation tapes (H1, H2, and H3) were established. The root mean square errors (nRMSE) of these three models were 0.66%, 0.74%, 0.34%, respectively, indicating their excellent prediction performance. The optimal performance of the three types of tapes were obtained when the fertilizer concentration was 0.6 g/L, the sediment content was 1 g/L, and the operating pressure was 40 kPa. Under the optimal condition, the average relative flows of H1-type, H2-type, and H3-type were 0.792, 0.764, and 0.700, respectively.

## Introduction

The average per capita water resource in the world is about 9000 m^3^, yet the number is only 2200 m^3^ in China. China is one of the water-deficient countries in the world. It is estimated that the population of China will reach 1.6 billion by 2030, which will cause the water shortage to increase by 40 to 60 billion m^3^^1^. Because drip irrigation can be controlled precisely, fertilizer and water can be directly transported to the root area. This process greatly reduces the use of irrigation water and fertilizer and improves the utilization of water resources. The last stage of the drip irrigation system is the drip irrigation tape, the hydraulic performance of which has a large impact on the performance, cost, and service life of the entire drip irrigation system^2^.

Whether good irrigation water quality or perfect filtration measures, the irrigation systems are still physical clogging by 31%^3^. Many factors affect the clogging of drip irrigation belts, such as manufacturing deviation, terrain deviation, flow channel structure, working pressure, laying slope, laying length, sand content, sand particle size, fertilizer type, fertilizer concentration, and irrigation water temperature^4–10^. In a study by Xu et al.^11^, muddy water with a sediment content of 1.0, 1.25, and 1.5 g/L was prepared to investigate the causes and influences of sediment content and operating pressure (25 kPa and 75 kPa) on the clogging of inserted labyrinth channel drippers. Ren et al.^12^ studied the clogging of a large-channel labyrinth dripper under conditions of continuous sediment addition and intermittent sediment addition, and they identified the influence of a sediment content of 2 g/L on dripper clogging and irrigation uniformity. Using eight different levels of sediment content (0.25–2.0 g/L), Niu et al.^13^ calculated the average flow rate and irrigation uniformity and analyzed the effect of sediment grain size and grain concentration on dripper clogging. Research on the influence of many factors on the anti-clogging performance of drip irrigation belts is not perfect. Fertilization changes the temperature, viscosity coefficient, solid particle content, pH value, electrical conductivity, and other parameters of the water source, causing various solutes to collide, adsorb, agglomerate, and precipitate under the action of turbulent water flow in the flow channel, increasing the risk of clogging^14–16^. In particular, urea affected the flocculation and sedimentation of sediment particles. Phosphorus fertilizer was likely to react with Ca^2+^ and Mg^2+^ in irrigation water to produce insoluble precipitate. Fertilizer containing Ca^2+^ and SO_4_^2−^ also greatly increased the probability of dripper clogging^17–19^. The order of influence on clogging was given as phosphorus fertilizer > potash fertilizer > urea > compound fertilizer. In addition, when the fertilization concentration was greater than 0.6 g/L, dripper clogging was significantly accelerated^20^. Fertilization also provides favorable conditions for the growth of microorganisms, and the microorganisms will be adsorbed on the sediment particles to accelerate the blockage of the dripper^21–24^. In recent years, the use of water-fertilizer integrated drip irrigation technology has been rapidly popularized, yet it also has aggravated the problem of dripper clogging^25^.

Mattar used artificial neural network (ANN) and gene expression programming model (GEP) to predict the emitter flow variation (q_var_) and the manufacturer’s coefficient of variation (CV_m_) of the emitter, by evaluated the influence of the structural parameters of different labyrinth-channel emitters on its hydraulic performance at different working pressures and water temperatures, and identified the important structural parameters of the labyrinth-channel emitters affecting q_var_ and CV_m_^26^. The approach of ANN and GEP needs hierarchical assumptions, critical value assumptions, neural network structure assumptions, data sifting and transformation. Xi explored the influence of the drip irrigation belt operating pressure and laying length on the irrigation uniformity, and established a mathematical regression model of the irrigation uniformity of two drip irrigation belts to find the optimal combination of lay length and operating pressure^27^. Simple regression models discard a lot of useful information about some variable data, so the residuals of the model are larger.

## Significance and novelty of the work

Projection pursuit regression (PPR) is an assumption-free modeling method, and the data do not need to meet the assumptions of normality and homogeneity of variance. The operation of PPR is simple and does not require exponential or logarithmic transformation of the data, and the model has high precision and accuracy^28^. The novelty of this study is that PPR modeling tool is used to establish the expression of the three factors and the average relative flow, so as to predict the optimal working conditions and rank the influence of fertilizer concentration, sediment content, and working pressure on the average relative flow. In this study, we analyzed three factors, i.e., fertilizer concentration, sediment content, and operating pressure, of three types of single-wing labyrinth drip irrigation tapes to investigate their influences on the average relative flow. We conducted an indoor test with a drip irrigation tape length of 35 m and obtained the order of influences of the three factors on the average relative flow. These findings provide a reference for the prediction of average relative flow of single-wing labyrinth drip irrigation tapes and provide the theoretical basis and technical support needed for the application of water-fertilizer integrated drip irrigation.

## Materials and methods

### Materials

**Figure 1:**
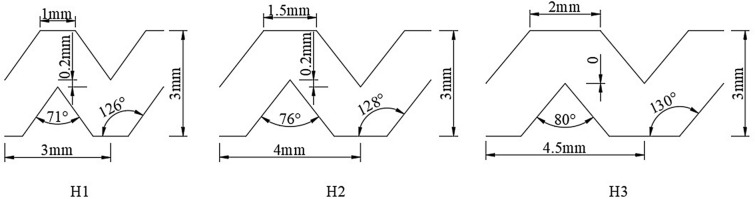
Photograph of drip irrigation tape.

A single-wing labyrinth drip irrigation belt produced by a water-saving irrigation company widely used in China was selected for experiments. The rated flow rates of the drip irrigation belts produced by this company are 1.8, 2.4, 2.6, 2.8, and 3.2 L/h, and drip irrigation belts with rated flow rates of 1.8, 2.6, and 3.2 L/h were selected for study. The structural parameters of these three types of single-wing labyrinth drip irrigation belts are shown in Fig. 1, and the hydraulic performance parameters are shown in Table 1. The most commonly used potassium-sulfate-type compound fertilizer was chosen, which has the advantages of good water solubility, easy absorption, high nutrient content, few auxiliary components, and good physical properties; the nutrient content was N: P_2_O_5_: K_2_O = 17: 17: 17, and total nutrient content was greater than 51%.

**Table 1:** Hydraulic performance parameters of studied drip irrigation tapes.

Type	Outer diameter (mm)	Water outlet spacing (cm)	Number of trapezoidal flow channel teeth	Rated pressure (kPa)	Rated flow (L/h)	Flow coefficient	Flow regime index
H1	16	30	85	100	1.8	0.11	0.60
H2	16	30	63	100	2.6	0.16	0.61
H3	16	30	54	100	3.2	0.25	0.55

**Figure 2:**
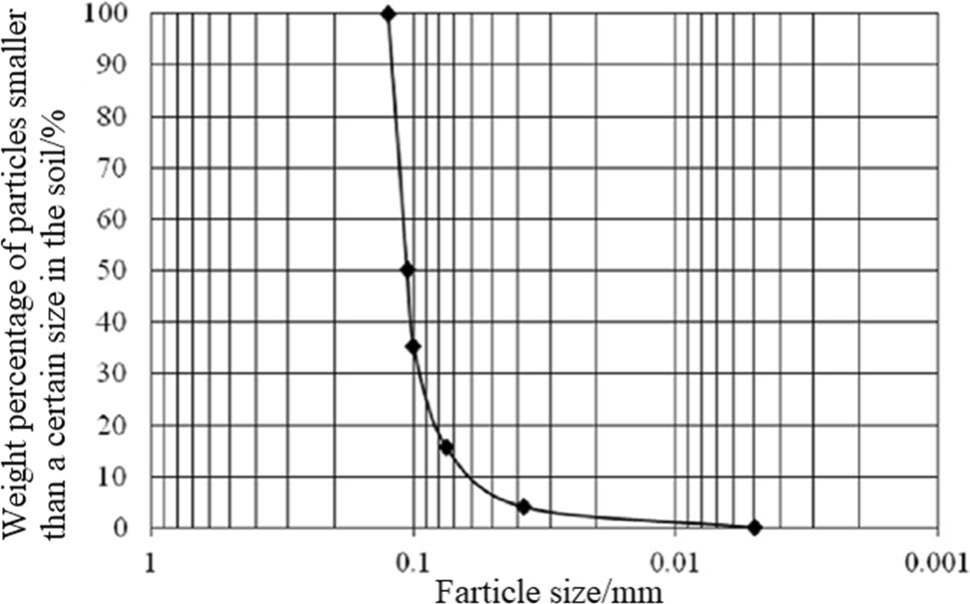
Sediment particle curve.

The natural soil from Xishan, Urumqi, China, was used as the sediment, which was passed through a 120-mesh sieve. Then, the particle size was larger than 0.074 mm, a set of standard-sieve to sieve. Collect the sieve balance of each sieve, and weigh the particles to obtain the percentage of soil weight. Finally, the particle size was smaller than 0.074 mm, configured into a soil suspension with uniform concentration was prepared with a density meter at different times, and suspension densities were measured. According to the densitometer reading and soil particle subsidence time, the percentage of soil weight of particles was calculated, and finally the distribution curve of particle size was drawn as shown in Fig. 2. The proportion of particles with a size < 0.125 mm was 100%, and the proportion with a size < 0.1 mm accounted for 35.28%. The median grain size D_50_ was 0.106 mm.

**Figure 3:**
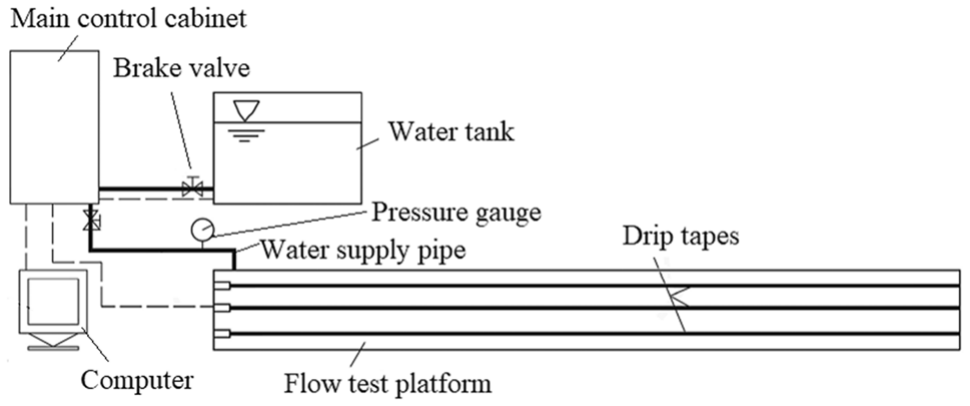
Schematic of anti-clogging performance test platform for drip irrigation tapes/pipes.

A schematic of the test platform is shown in Fig. 3. This set of drip irrigation belt anti-clogging performance test bench models was KD-DJC, manufactured by Hebei Kedao Testing Machine Technology Co., Ltd., and the system is suitable for a voltage of 380 V. The main control cabinet includes a Xilin SD200 vector inverter, the highest frequency is 0–600 Hz, the load frequency is 2–10 kHz, and the speed regulation range is 1:50 or 1 Hz/150% rated torque. A 32CDLF4-150 light multi-stage pump, produced by Yongjia Yingke Pump Valve Co., Ltd., with a flow of 4 m^3^/h, speed of 2880 rpm, lift of 120 m, and power of 3 kW, was used. Also employed was a YE2-802-2 three-phase asynchronous motor, with a power of 11 kW, voltage of 380 V, frequency of 50 Hz, rotation speed of 2830 rpm. An IRK50-100A centrifugal pump with a flow rate of 22.3 m^3^/h, lift of 10 m, matching power of 1.1 kW, and rotation speed of 2900 rpm was also employed. The length of the drip irrigation tape test platform was 35 m.

### Introduction to projection pursuit regression

PPR is a new and valuable new technology developed by the international statistical community in the mid-1970s. It is an interdisciplinary subject of statistics, applied mathematics, and computer technology. It uses computer technology to project high-dimensional data onto a low-dimensional subspace and finds a projection that can reflect the structure and characteristics of high-dimensional data by minimizing a certain projection index. It has the advantages of robustness, anti-interference, and high accuracy, so it is widely used in many fields. It has been applied to nonlinear function approximation and function smoothing, as well as to principal component and independent component analysis. It offers advantages in extracting the internal structural characteristics of high-dimensional data (e.g., influencing factors and degree of influence). Moreover, the PPR model has a higher accuracy than other models. In the PPR model, the sum of a series of ridge functions is used to approximate a regression function. The key to PPR is to estimate *f*_*i*_ and to determine the optimal combination of *α*_*ij*_ and *β*_*i*_. If *x* is a *P*-dimensional independent variable and *y* the dependent variable^23^, then

1 $$\widehat{y} = E\left(y|x\right) = \overline{y }+\sum_{i = 1}^{M}{\beta }_{i}{f}_{i}\left(\sum_{j = 1}^{P}{\alpha }_{ij}{x}_{ij}\right)$$

where *Ef*_*i*_ = 0, *Ef*_*i*_^*2*^ = 1, $\sum_{j = 1}^{P}{{\alpha }_{ij}^{2}}$=1, *f*_*i*_ is the *i*th ridge function, *M* and *M*_*u*_ are the upper limit and the optimal number of ridge functions, *β*_*i*_ is the weight coefficient of a ridge function, *α*_*ij*_ is the *i*th component in the *jth* direction, and *Q* is the number of dependent variables.

The steps of solving the PPR model are the following:

1. Select the initial projection direction α.
2. Perform a linear projection on ${\left\{{X}_{i}\right\}}_{j}^{n}$ to obtain ${\alpha }^{T}{X}_{i}$, and perform a smoothing method on $\left({\alpha }^{T}{X}_{i}, {Y}_{i}\right)$ to determine the ridge function ${f}_{\alpha }\left({\alpha }^{T}\right)X, i=1,\dots ,n$.
3. Set the value of ${\sum_{i=1}^{n}\left({y}_{i}-{f}_{\alpha }\left({\alpha }^{T}{X}_{i}\right)\right)}^{2}$ that minimizes α as α_1_. Repeat Step (2) until the error does not change, and then determine α_1_ and ${f}_{1}\left({\alpha }_{1}^{T}X\right)$.
4. Let the fitting residual of ${r}_{1}\left(X\right)=Y-{f}_{1}\left({\alpha }_{1}^{T}X\right)$ obtained from the first calculation replace Y. Repeat Steps (1)–(3) to obtain α_2_ and ${f}_{2}\left({\alpha }_{2}^{T}X\right)$.
5. Repeat Step (4). Calculate ${r}_{2}\left(X\right)={r}_{1}\left(X\right)-{f}_{2}\left({\alpha }_{2}^{T}X\right)$ to replace ${r}_{1}\left(X\right)$ until the *M*th ${\alpha }_{M}$ and ${f}_{M}\left({\alpha }_{M}^{T}X\right)$ are obtained and $\sum_{i=1}^{n}{r}_{i}^{2}$ no longer decreases or a certain accuracy criterion is met.
6. Determine the last *m*th α and f.
7. Calculate $$\mathrm{f}\left(\mathrm{x}\right)=\sum_{\mathrm{m}-1}^{\mathrm{M}}{\mathrm{f}}_{\mathrm{m}}\left({\mathrm{\alpha }}_{\mathrm{m}}\mathrm{X}\right)$$.

### Uniform orthogonal test

According to the actual situation of irrigation water, the designed sediment content is 1, 2, and 3 g/L^29^. Three levels, i.e., 0.6, 1.8, and 3 g/L, were designed according to the actual irrigation fertilizer concentration. Low-pressure and small-flow drip irrigation technology is the most advanced drip irrigation technology at present. It has the characteristics of low working pressure, low operating energy consumption, low engineering investment, and good irrigation uniformity^30^. In this study, three gradients of operating pressures, i.e., 40, 70, and 100 kPa, were designed. The uniform orthogonal design table UL_9_ (3^3^) for the three factors of fertilizer concentration, sediment content, and working pressure (marked E, S, and B, respectively) was used, as shown in Table 2.

**Table 2:** UL_9_ (3^3^) uniform orthogonal test design.

No	Factor		
	*E* fertilizer concentration (g/L)	*S* sediment content (g/L)	*B* operating pressure (kPa)
1	0.6	1.0	40
2	0.6	2.0	100
3	0.6	3.0	70
4	1.8	1.0	100
5	1.8	2.0	70
6	1.8	3.0	40
7	3.0	1.0	70
8	3.0	2.0	40
9	3.0	3.0	100

## Results and discussion

### Test results and model establishment

Twenty-five drippers for each drip irrigation tape were chosen, and a 1000-mL water collection bucket was placed under them to collect water. To accelerate the test process, referring to the draft international dripper anti-clogging research standard, the irrigation period was shortened in equal proportions^31^. The selected irrigation time was 10 min, and the irrigation interval was 30 min. A total of nine irrigations were performed. After the irrigation was completed, two 15 min flow measurements were performed, and the average value taken. The dripper flow adopts the weighing method, and a model YP200N electronic balance (Shanghai Jinghai Instrument Co., Ltd., China) was used. Its maximum weight capacity is 2000 g and the division value is 0.01 g. Before the muddy water test started, clean water was added to the cleaned muddy water tank and the clean water flow measured under the current conditions. After the clean water test, the original arrangement was kept unchanged, and prepared sandy water with the corresponding concentration was added to the muddy water tank for flow measurement. After each group of treatments, the drip irrigation belt was replaced with a new one, and the system pipes, water tanks, and pumps were flushed.

In this study, the clogging of the drip irrigation system was determined based on the average relative flow, and the threshold was set to 0.75^11^. The equation for the calculation of average relative flow is

2 $$q = \frac{\sum_{1}^{N}\frac{{q}_{pi}}{{q}_{i}}}{N}$$

where *q* is the average relative flow, *i* is the number of a dripper, *N* is the total number of drippers, *q*_*pi*_ is the flow of the *i*th dripper in muddy water (L/h), and *q*_*i*_ is the flow of the *i*th dripper in clean water (L/h).

The test was carried out strictly according to the test table of uniform orthogonal design, and the average relative flow under each muddy water condition calculated by Eq. (2); see Table 3 for details. A PPR model was established to analyze the 27 groups of average relative flow data in Table 3. The smooth coefficient of the projection sensitivity was 0.5, and the number of projections M was 5. Because Mu should be smaller than M, we set Mu to 3. The final modeling parameters were as follows: N = 9, P = 3, Q = 1, M = 5, Mu = 3.

**Table 3:** Results of uniform orthogonal tests.

No	Factor level			Average relative flow (q)		
	*E* fertilizer concentration (g/L)	*S* sediment content (g/L)	*B* operating pressure (kPa)	H1	H2	H3
1	0.6	1	40	0.793	0.702	0.764
2	0.6	2	100	0.653	0.628	0.653
3	0.6	3	70	0.564	0.523	0.587
4	1.8	1	100	0.702	0.693	0.711
5	1.8	2	70	0.564	0.657	0.588
6	1.8	3	40	0.523	0.502	0.534
7	3	1	70	0.603	0.647	0.621
8	3	2	40	0.55	0.573	0.548
9	3	3	100	0.431	0.457	0.451

Through PPR analysis, we obtained the weight coefficient β of the ridge function and the projection direction α of H1, H2, and H3, as in Eqs. (3)–(8). The vector formula of β and α of each factor were substituted into Eq. (1) to obtain the final calculation model, as follows:

3 $$\beta = \left(0.992, 0.140, 0.063\right)$$

4 $$\left(\begin{array}{c}{\mathop{\alpha }\limits^{\rightharpoonup} }_{1}\\ {\mathop{\alpha }\limits^{\rightharpoonup} }_{2}\\ {\mathop{\alpha }\limits^{\rightharpoonup} }_{3}\end{array}\right) = \left(\begin{array}{ccc}-0.520& 0.489& 0.251\\ -0.854& 0.872& -0.968\\ -0.003& -0.040& -0.011\end{array}\right)$$

5 $$\beta = \left(1.018, 0.150, 0.075\right)$$

6 $$\left(\begin{array}{c}{\mathop{\alpha }\limits^{\rightharpoonup} }_{1}\\ {\mathop{\alpha }\limits^{\rightharpoonup} }_{2}\\ {\mathop{\alpha }\limits^{\rightharpoonup} }_{3}\end{array}\right) = \left(\begin{array}{ccc}-0.496& -0.124& 0.296\\ -0.868& 0.992& 0.954\\ -0.001& -0.028& 0.052\end{array}\right)$$

7 $$\beta = \left(0.987, 0.234, 0.280\right)$$

8 $$\left(\begin{array}{c}{\mathop{\alpha }\limits^{\rightharpoonup} }_{1}\\ {\mathop{\alpha }\limits^{\rightharpoonup} }_{2}\\ {\mathop{\alpha }\limits^{\rightharpoonup} }_{3}\end{array}\right) = \left(\begin{array}{ccc}-0.253& 0.489& 0.251\\ -0.967& 0.872& -0.968\\ 5.760& 0.002& -0.023\end{array}\right)$$

### Influences of the factors on average relative flow

The results of variance analysis are shown in Table 4. At a 95% confidence interval, the influences of fertilizer concentration and sediment content on the average relative flow of the H1 drip irrigation tape were significant, the influence of sediment content for the H2 drip irrigation tape was significant, and the influence of fertilizer concentration was important. For the H3 drip irrigation tape, the influence of sediment content was significant, and the influence of fertilizer concentration was important. In addition, the influence of operating pressure for none of the three types of drip irrigation tapes was crucial. These results suggested that fertilizer concentration and sediment content had a substantial influence on the relative flow of the dripper and were the key influencing factors of dripper clogging. In terms of the fertilizer concentration, its influence on the relative flow of H1 was significant, and the influence for H2 and H3 only reached the substantial level. Although all three types of drip irrigation tapes had single-wing labyrinth channels, H1 had a narrower channel compared with H2 and H3, which promoted the collision and flocculation of sediment particles to a certain extent, leading to a higher probability of stable agglomeration formation and dripper clogging. On the basis of the *P* values, the order of the influences of the factors on the average relative flow was sediment content > fertilizer concentration > operating pressure.

**Table 4:** Variance analysis of average relative flow.

Type	Source of variance	Sum of squares	Degree of freedom	Mean square	F	*P*
H1	Fertilizer concentration	0.030	2	0.015	121.908	0.008
	Sediment content	0.056	2	0.028	227.378	0.004
	Operating pressure	0.003	2	0.002	12.377	0.075
	Error	0.000	2	0.000		
H2	Fertilizer concentration	0.007	2	0.003	25.732	0.037
	Sediment content	0.054	2	0.027	204.190	0.005
	Operating pressure	0.001	2	0.000	2.048	0.328
	Error	0.0003	2	0.0001		
H3	Fertilizer concentration	0.025	2	0.012	66.928	0.015
	Sediment content	0.046	2	0.023	125.351	0.008
	Operating pressure	0.000	2	0.000	1.152	0.465
	Error	0.0004	2	0.0002		

*P < 0.05, **P < 0.01.

Moreover, the weight coefficient of the ridge function of each factor was calculated according to PPR, and the results are shown in Table 5. Based on the weights of the factors, the order of influences for H1, H2 and H3 is as follows: sediment content > fertilizer concentration > operating pressure. These results were consistent with the results of the variance analysis.

**Table 5:** Weight coefficients of influencing factors.

Type	Factor	Sediment content (g/L)	Fertilizer concentration (g/L)	Operating pressure (kPa)
H1	Weight coefficient	1	0.712	0.261
H2		1	0.733	0.076
H3		1	0.512	0.185

To summarize, the order of factors affecting the average relative flow of the single-wing labyrinth drip irrigation belt is sand content > chemical fertilizer concentration > working pressure. Sediment content is the main factor affecting the blockage of drip irrigation belts. It was found that particles with a particle size in the range 0.034–0.067 mm are difficult to flow out of the channel^32^, and the sediment in this range will form a film on the wall of the flow channel^33^. This will lead to a decrease in the average relative flow, and measures should be taken to reduce the sediment content in this particle size range during muddy water irrigation with integrated water and fertilizer. Fertilizer concentration is the second influencing factor. Potassium sulfate has an inhibitory effect on the ability of the dripper to transport sand^34^. It is easy to form the precipitation of sulfate through application of potassium sulfate fertilizer, and the adsorption of the precipitation on the wall of the capillary will increase the roughness of the wall of the drip irrigation belt. This will increase the probability of sediment particles colliding, thereby slowing the flow of water and making sediment particles more likely to settle^35,36^. Regarding sediment content > fertilizer concentration, fertilization will change the cation concentration in water, whereas most fine-grained sediments are negatively charged, and the cations compress the electric double layer structure on the surface of the sediment by neutralizing^37^. As a result, the electrostatic repulsion between sediment particles is reduced, which enhances flocculation of sediment, making it easier for the sediment to form a stable agglomeration structure to block the dripper^38^. When the actual sand content is large, the fertilization concentration should be reduced to reduce the occurrence of clogging.

### PPR model evaluation

The error of the measured and predicted values of the average relative flow calculated and analyzed by the PPR program is shown in Table 6. The maximum relative errors of the average relative flow of H1, H2, and H3 were 1.24%, 1.07%, and 0.94%, respectively. When we used an absolute relative error of under 5% to calculate the pass rate of the modeling samples, the pass rate of nine testing datasets was 100%.

**Table 6:** Results of PPR models.

Type	Group	Measured value	Predicted value	Absolute error	Relative error (%)
H1	1	0.793	0.792	− 0.001	0.164
	2	0.653	0.656	0.003	0.490
	3	0.564	0.558	− 0.006	1.082
	4	0.702	0.698	− 0.004	0.527
	5	0.564	0.565	0.001	0.230
	6	0.523	0.526	0.003	0.574
	7	0.603	0.611	0.008	1.244
	8	0.55	0.549	− 0.001	0.200
	9	0.431	0.428	− 0.003	0.650
H2	1	0.764	0.764	− 0.001	0.065
	2	0.653	0.660	0.007	1.072
	3	0.587	0.584	− 0.003	0.545
	4	0.711	0.704	− 0.007	1.055
	5	0.588	0.586	− 0.002	0.323
	6	0.534	0.539	0.005	0.899
	7	0.621	0.626	0.005	0.741
	8	0.548	0.544	− 0.004	0.730
	9	0.451	0.452	0.001	0.133
H3	1	0.702	0.700	− 0.002	0.342
	2	0.628	0.629	0.001	0.175
	3	0.523	0.522	− 0.001	0.249
	4	0.693	0.693	0.000	0.058
	5	0.657	0.655	− 0.002	0.244
	6	0.502	0.507	0.005	0.936
	7	0.647	0.648	0.001	0.139
	8	0.573	0.572	− 0.001	0.262
	9	0.457	0.457	0.000	0.088

The three types of single-wing labyrinth drip irrigation belts with different flow rates have similar properties, and the amount of PPR modeling analysis data is large. Therefore, the H1-type drip irrigation belt was used to test the accuracy of the established PPR average relative flow prediction model. Nine groups of test samples were selected, and the comparison between the measured and predicted average relative flows is shown in Table 7. It can be seen from the table that the difference between the predicted value of the reserved test sample and the measured value was small, and the maximum relative error was 4.87%. When the absolute value of the relative error of under 5% was used to judge the pass rate of each modeling sample, the pass rate of the nine testing datasets was 100%.

**Table 7:** Comparison of measured and predicted values of average relative flow of testing samples (H1 type).

No	Factor level			Average relative flow		Error	
	*E* fertilizer concentration (g/L)	*S* sediment content (g/L)	*B* operating pressure (kPa)	Measured value	Predicted value	Absolute error	Relative error (%)
1	0.6	0.5	40	0.836	0.801	− 0.035	4.187
2	0.6	1.5	100	0.743	0.719	− 0.024	3.230
3	0.6	2.5	70	0.572	0.594	0.022	3.846
4	1.8	0.5	100	0.734	0.766	0.032	4.360
5	1.8	1.5	70	0.657	0.625	− 0.032	4.871
6	1.8	2.5	40	0.534	0.559	0.025	4.682
7	3	0.5	70	0.667	0.674	0.007	1.049
8	3	1.5	40	0.608	0.593	− 0.015	2.467
9	3	2.5	100	0.438	0.457	0.019	4.338

The root mean square error (RMSE) is described as the root of the mean square error: the proportion of the sum of the square of the difference between estimated and actual values to the total number of observations. The equations are^39^

9 $$RMSE = \sqrt{\frac{\sum_{i = 1}^{n}{\left({A}_{i}-{F}_{i}\right)}^{2}}{n}}$$

10 $$nRMSE = \frac{RMSE}{\overline{A} }\times 100\%$$

where A_*i*_ is the actual value, F_*i*_ is the estimated value, *n* is the number of samples, RMSE is the root mean square error, $\overline{A }$ is the average value of the actual data, and nRMSE is the ratio of the RMSE to the average value. To evaluate the performance of the models, the following criteria were introduced: (1) nRMSE < 10%, excellent; (2) 10% < nRMSE < 20%, good; (3) 20% < nRMSE < 30%, fair; and (4) nRMSE > 30%, poor^26^.

According to Eqs. (9) and (10), the average relative flow nonlinear model nRMSE of H1, H2, and H3 drip irrigation belts are 0.66%, 0.74%, and 0.34%, respectively, all of which less than 10%. In conclusion, the performance of the established PPR average relative flow model under muddy water conditions is excellent, indicating that the established PPR model has good stability and high accuracy.

### Model simulation and optimization

**Figure 4:**
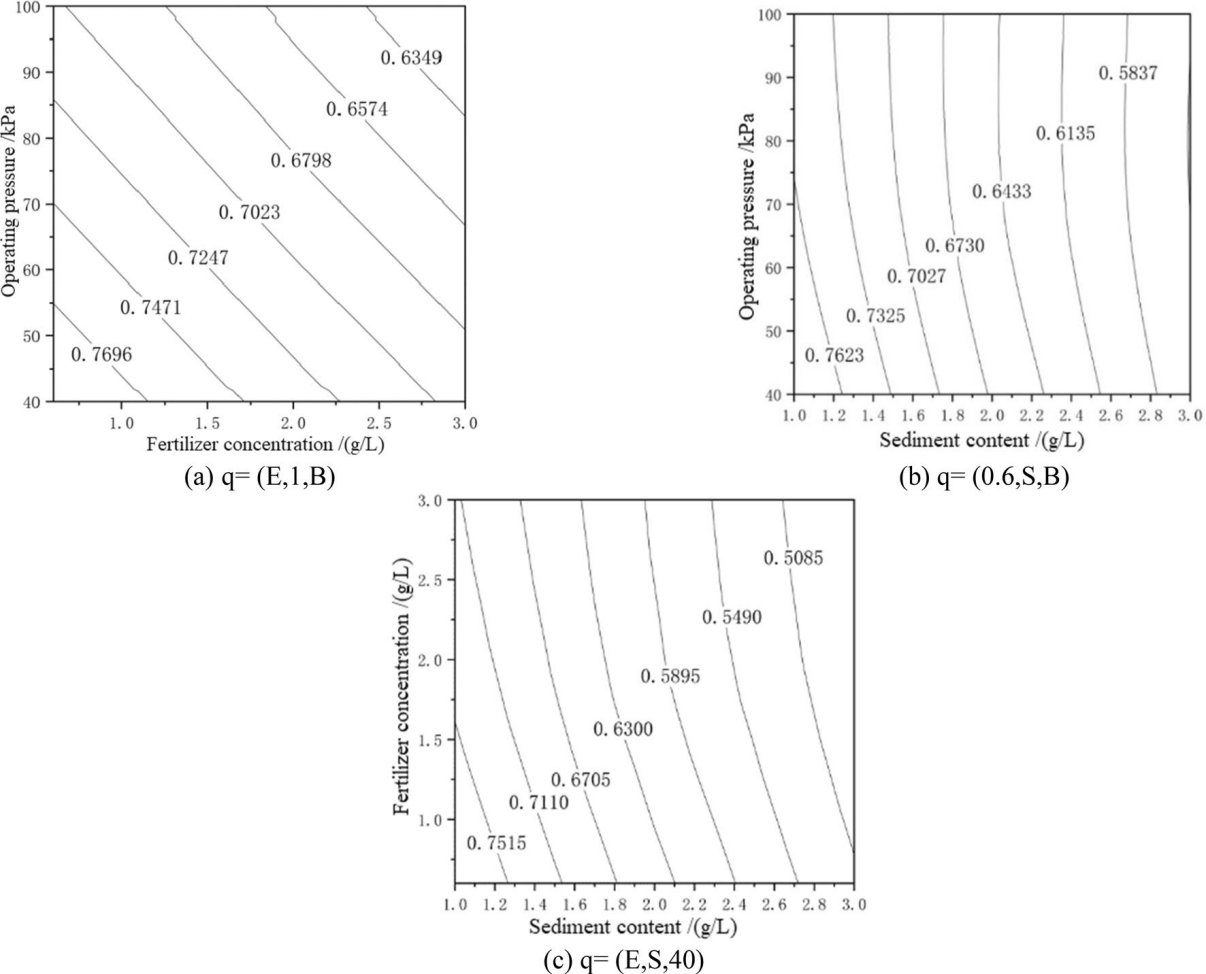
PPR contour map for H1 drip irrigation tape.

Once the order of influence of the factors (i.e., fertilizer concentration, sediment content, and operating pressure) on the average relative flow was obtained, we then used the PPR model to find the internal structure of the data and calculated the average relative flow under different conditions. By fixing one factor and changing the other two, a contour plot of the average relative flow was obtained, as shown in Fig. 4.

In Fig. 4a, the average relative flow rate of the H1-type drip irrigation tape was reduced by 17.5%. It can be seen that under the condition of sand content of 1 g/L the average relative flow rate decreases faster in the range 40–100 kPa. Under the condition that the sand content remains unchanged, the increase in the fertilizer concentration can promote the clogging of the dripper. In Fig. 4b, the average relative flow decreased by 23.4%. In Fig. 4c, the average relative flow decreased by 32.34%, and the average relative flow decreased by 5.39% in the chemical fertilizer concentration range 0.6–3.0 g/L. Under the condition of pressure of 40 kPa, the effect of sediment content on the average relative flow is more significant than that of fertilizer concentration. From the comprehensive graph and analysis, it can be seen that the optimal operating conditions are as follows: the chemical fertilizer concentration is 0.6–1.2 g/L, the sand content is 1 g/L, and the value range of working pressure is 40–55 kPa.

**Figure 5:**
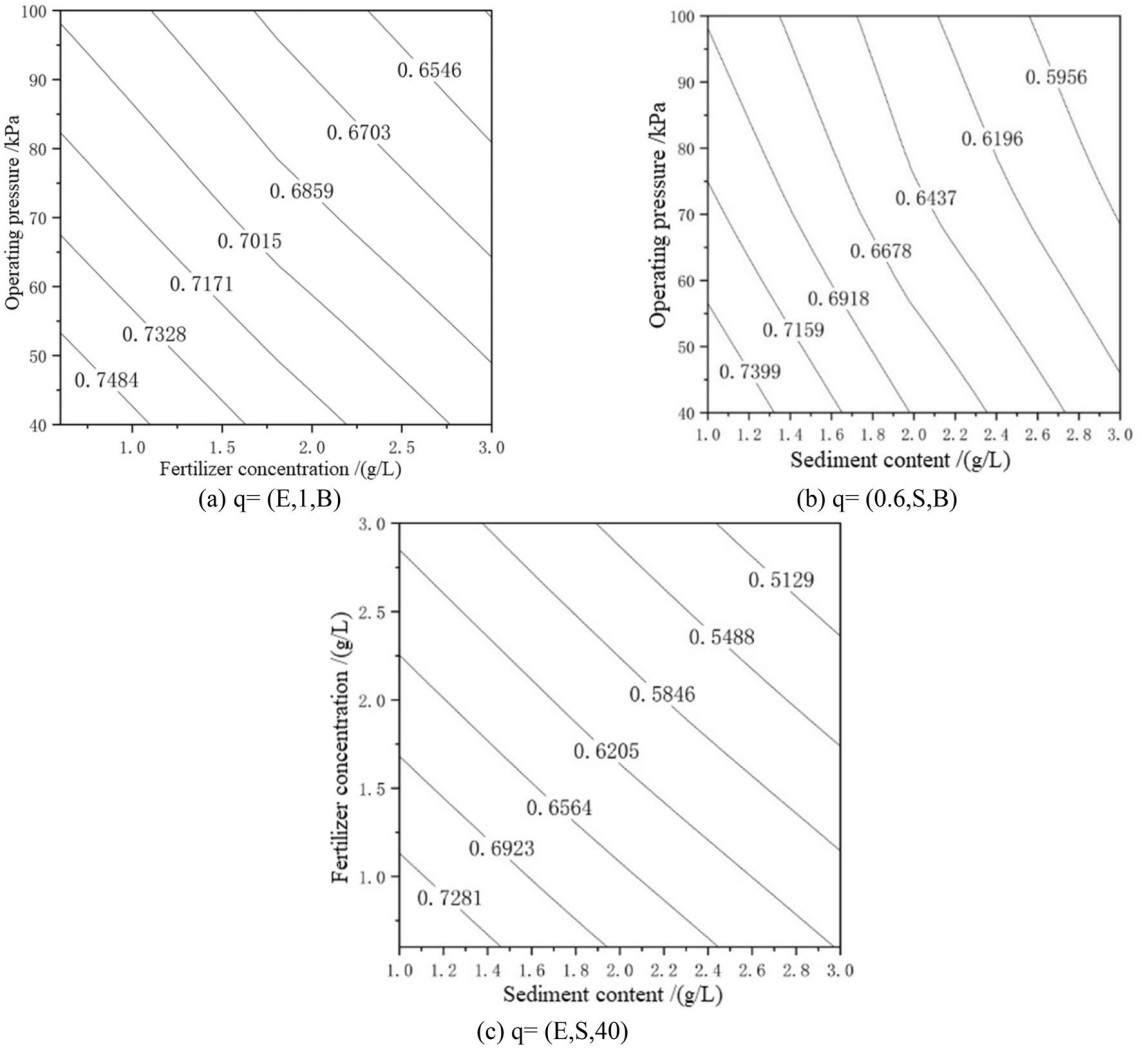
PPR contour map for H2 drip irrigation tape.

In Fig. 5a, the average relative flow rate of the H2-type drip irrigation tape decreased by 12.53%. It can be seen that under the condition of 1-g/L sediment content, the pressure change in the range 40–100 kPa and the flow rate drop in the range of chemical fertilizer concentration from 0.6 to 3 g/L are smaller. In Fig. 5b, under the condition of a chemical fertilizer concentration of 0.6 g/L, the average relative flow decreased rapidly with increasing sediment content, and the decrease was 15.51%. In the pressure range 40–100 kPa, the average relative flow changed little, with a decrease of 6.02%. In Fig. 5(c), under the working pressure of 40 kPa, the average relative flow rate decreased by 29.6%. From the comprehensive graph and analysis, it can be seen that the optimal operating conditions are as follows: the chemical fertilizer concentration is 0.6–1.2 g/L, the sand content is 1 g/L, and the value range of working pressure is 40–55 kPa.

**Figure 6:**
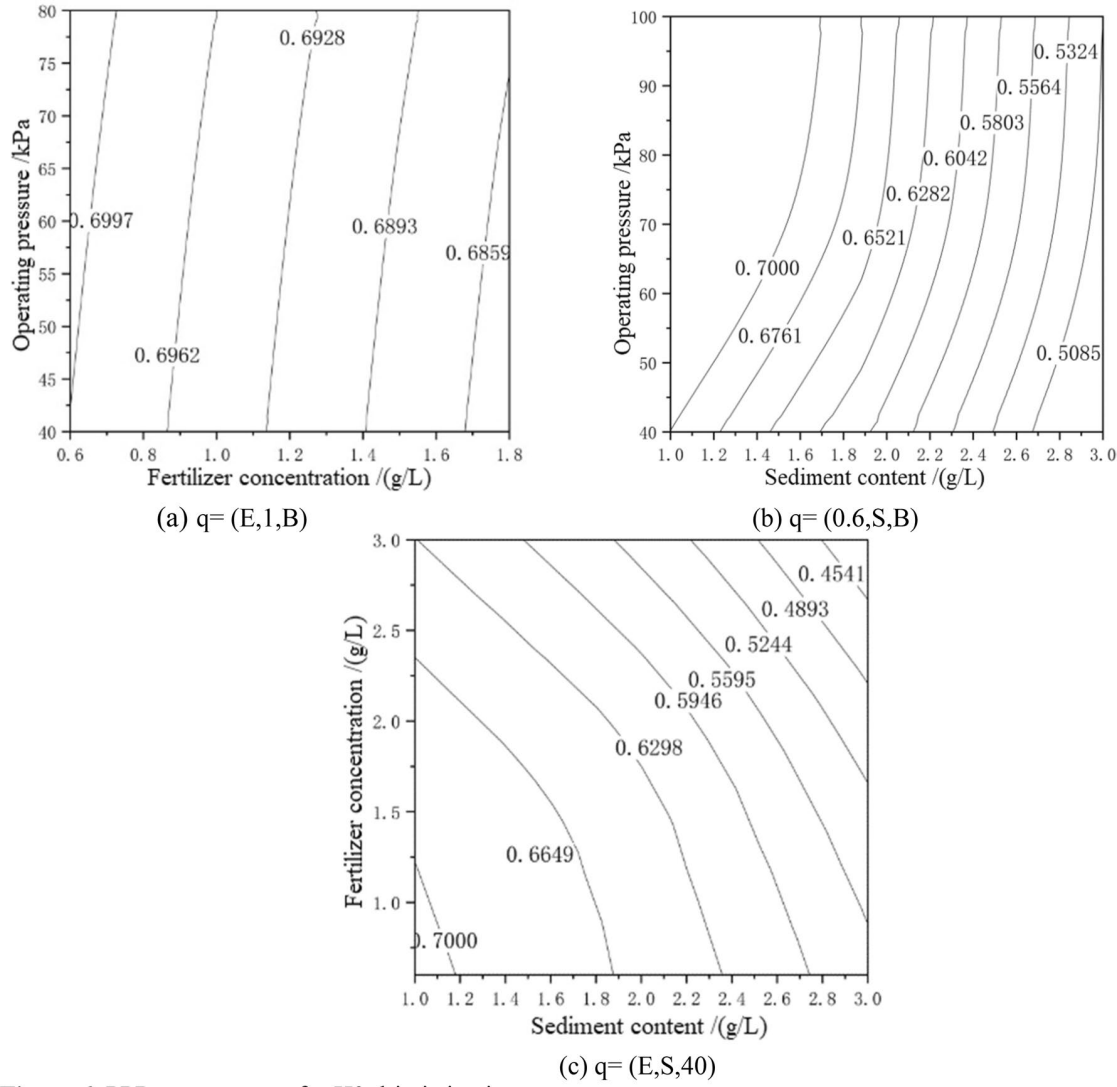
PPR contour map for H3 drip irrigation tape.

In Fig. 6a, the average relative flow of the H3-type drip irrigation tape decreased by 1.97%. It can be seen from Fig. 6b that under the condition of a chemical fertilizer concentration of 0.6 g/L the average relative flow rate decreases rapidly with increasing sediment content, i.e., by 28.57%. The maximum average relative flow rate is reached in the range of sediment content of 1.0–1.6 g/L. Under the condition of a chemical fertilizer concentration of 0.6 g/L, the pressure change has no effect on the average relative flow. In Fig. 6c, the average relative flow decreased by 35.13%. The average relative flow rate decreased by 10.03% in the range of pressure fertilizer concentration of 0.6–3.0 g/L. Under the condition of pressure of 40 kPa, the effect of sediment content on the average relative flow is more significant than that of chemical fertilizer concentration. From the comprehensive graph and analysis, it can be seen that the optimal operating conditions are as follows: of the chemical fertilizer concentration is 0.6–1.2 g/L, the pressure is 40 kPa, and the sand content is 1.0–1.2 g/L.

To summarize, there is an optimal combination of three factors in the ranges of chemical fertilizer concentration of 0.6–1.2 g/L, of sand content of 1.0–1.2 g/L, and of working pressure of 40–55 kPa. The calculation results of the PPR optimization simulation are shown in Table 8.

**Table 8:** Optimal combination of average relative flow.

Type	S sediment content (g/L)	E fertilizer concentration (g/L)	B operating pressure (kPa)	Measured q value	Predicted q value
H1	1	0.6	40	0.793	0.792
H2	1	0.6	40	0.764	0.764
H3	1	0.6	40	0.702	0.700

When the average relative flow rate is set to 0.75 to judge whether blockage occurs, it can be seen from the analysis of the average relative flow PPR model that the H1-type and H2-type have not yet reached the blockage standard, while the H3-type has reached a serious degree of blockage. In the actual project, the high sediment content is concentrated in the flood season^29^. In this experiment, under the irrigation conditions of high sediment content and high chemical fertilizer concentration, the clogging standard is still not reached after the ninth irrigation. Therefore, actual irrigation conditions are more difficult than the test conditions. When the muddy water sediment content is not less than 1 g/L, the fertilizer concentration should be controlled at approximately 0.6 g/L, which is consistent with Li’s research results^38^. When the control working pressure is 40 kPa, the rated flow of the H1-type is the smallest (1.8 L/h) among the three types of drip irrigation belts, which indicates the technical feasibility of low pressure and small flow. In addition, the maximum average relative flow can be obtained, which greatly reduces the engineering and operation costs of drip irrigation, facilitating the promotion and application of drip irrigation technology.

According to the PPR contour map in Figs. 4, 5, and 6, the three influencing factors of the average relative flow of the three drip irrigation belts can also be ranked. These results were consistent with the results of the variance analysis and the weight coefficient of a ridge function. The flow index of H1-type, H2-type and H3-type are 0.60, 0.61 and 0.55, respectively. The smaller the operating pressure sensitivity, the smaller the flow index, and the better the hydraulic performance. From the PPR contour map, it can be seen that H1-type and H2-type have little difference in sensitivity to operating pressure, and H3-type is the least sensitive to operating pressure, so H3-type has the best hydraulic performance. It can be seen from Figs. 4, 5 and 6 that the average relative flow of H1-type, H2-type and H3-type are negatively correlated with the concentration of chemical fertilizers, and the average relative flow of H1-type and H2-type have a negative correlation with operating pressure and sand content. But, the average relative flow of the H3-type is positively correlated with the operating pressure and sand content, which is caused by the difference in structure. The flow coefficients of H1-type, H2-type and H3-type are 0.11, 0.16, and 0.25, respectively. The better the combination of structural parameters, the greater the flow coefficient, and the better the hydraulic performance. According to the analysis, the sensitivity of H1-type, H2-type and H3-type to fertilizer concentration and sediment content is ranked as H1-type > H2-type > H3-type. The tooth spacing and the number of runner units have a positive correlation with the flow coefficient^40^. The tooth spacing and the number of runner units of H3-type are the largest, so the hydraulic performance of H3-type is better. The rated flow rates of H1-type, H2-type and H3-type are 1.8, 2.6 and 3.2 g/L respectively, and the flow rate is positively correlated with the flow coefficient.

To sum up, in the case of low pressure, low chemical fertilizer concentration, and low sand content, the H1-type is selected, which is conducive to the promotion of low pressure and small flow technology. In the case of high pressure, high chemical fertilizer concentration and high sand content, H3-type should be used to avoid blockage.

## Conclusions

In this study, with a fertilizer concentration in the range 0.6–3.0 g/L, sediment content in the range 1–3 g/L, and pressure in the range 40–100 kPa, we studied the influences of the three factors on the average relative flow of single-wing labyrinth drip irrigation tapes. We analyzed the results using variance analysis and PPR. The main conclusions are the following.

In the case of low pressure, low chemical fertilizer concentration, and low sand content, the H1-type is selected, which is conducive to the promotion of low pressure and small flow technology. In the case of high pressure, high chemical fertilizer concentration and high sand content, H3-type should be used to avoid blockage.

For the H1-type single-wing labyrinth drip irrigation tape, the influences of fertilizer concentration and sediment content on the average relative flow were significant. For the H2-type and H3-type, the influences of fertilizer concentration and sediment content on the average relative flow were significant. Moreover, the operating pressure did not significantly affect the average relative flow of all three types of drip irrigation tapes under the tested conditions. The order of influence of the factors is sediment content > fertilizer concentration > working pressure.

A PPR prediction model for fertilizer concentration, sediment content, working pressure, and average relative flow was constructed, and the model performed well. The combination of optimal factor levels for H1-type, H2-type, and H3-type are a fertilizer concentration of 0.6 g/L, sand content of 1 g/L, and a working pressure of 40 kPa.

## Supplementary Information

Supplementary Information.

## Data Availability

The data that supports the findings of this study are available in the supplementary material of this article.
