# Supplementary material for: Average relative flow of single-wing labyrinth drip irrigation tape based on projection pursuit regression
Source: Sci Rep. 2022 May 20;12:8543. doi: 10.1038/s41598-022-12638-y (PMC9123216; doi:10.1038/s41598-022-12638-y)
Supplement: Supplementary file 1 — Supplementary Information. [file 41598_2022_12638_MOESM1_ESM.pdf]

Table 1. Mass of 1000-ml collection bucket.

No.	H1-type (g)	No.	H2-type (g)	No.	H3-type (g)
1	87.05	26	86.00	51	86.90
2	87.00	27	86.70	52	86.90
3	87.10	28	87.00	53	86.90
4	87.00	29	85.05	54	86.90
5	87.00	30	86.20	55	87.00
6	86.90	31	85.20	56	87.00
7	86.70	32	85.20	57	87.00
8	86.90	33	87.00	58	87.00
9	87.00	34	85.10	59	86.90
10	86.60	35	87.00	60	86.90
11	87.10	36	85.90	61	87.00
12	86.80	37	86.30	62	86.90
13	86.70	38	85.70	63	86.90
14	87.00	39	85.50	64	86.90
15	87.10	40	85.50	65	87.00
16	87.00	41	85.20	66	86.90
17	87.05	42	85.60	67	87.00
18	86.70	43	85.50	68	86.80
19	87.00	44	85.10	69	87.00
20	86.80	45	85.10	70	87.00
21	86.90	46	85.10	71	86.90
22	87.00	47	85.10	72	87.00
23	87.00	48	87.10	73	86.90
24	86.70	49	85.40	74	87.00
25	86.90	50	85.10	75	87.00

Table 2. Mass of the collection bucket for the clean water test with the operating pressure of 40 kPa.

No.	H1-type (g)		No.	H2-type (g)		No.	H3-type (g)	
1	328.56	326.76	26	444.94	453.15	51	616.37	610.69
2	319.49	317.08	27	454.45	461.31	52	601.90	600.66
3	322.98	317.44	28	453.98	452.84	53	617.77	614.38
4	323.63	326.39	29	448.40	444.05	54	603.43	602.68
5	327.23	329.99	30	459.30	452.50	55	615.56	620.52
6	318.08	325.05	31	446.02	437.87	56	614.17	620.19
7	317.75	318.82	32	439.05	436.99	57	612.22	614.51
8	321.58	326.56	33	452.15	447.57	58	616.97	609.94
9	329.17	321.51	34	454.78	456.85	59	615.89	611.41
10	341.82	334.45	35	443.10	451.28	60	612.70	608.83
11	340.52	337.95	36	432.67	438.82	61	620.95	618.74
12	337.54	332.52	37	447.80	450.85	62	620.46	624.22
13	353.87	349.16	38	472.12	464.03	63	634.81	629.28
14	364.23	358.04	39	462.54	456.94	64	648.38	640.23
15	361.57	357.25	40	466.42	462.60	65	627.65	631.57
16	353.52	355.30	41	468.02	470.88	66	640.74	644.31
17	348.18	353.54	42	458.68	463.96	67	625.60	629.89
18	357.05	357.87	43	470.67	477.68	68	630.12	626.68
19	357.24	364.43	44	477.07	478.33	69	636.54	628.29
20	358.92	351.19	45	469.99	465.34	70	634.84	629.81
21	357.56	350.40	46	460.91	458.70	71	628.16	635.70
22	342.70	339.07	47	471.11	469.55	72	631.73	638.09
23	341.32	339.26	48	483.96	481.40	73	622.71	628.96
24	344.93	342.85	49	467.18	468.41	74	620.23	626.44
25	337.57	341.65	50	461.29	454.33	75	625.46	620.32

Table 3. Mass of the collection bucket for the clean water test with the operating pressure of 70 kPa.

No.	H1-type (g)		No.	H2-type (g)		No.	H3-type (g)	
1	428.32	429.81	26	587.97	594.92	51	797.74	803.11
2	436.13	438.00	27	590.36	592.38	52	803.87	806.47
3	427.29	433.13	28	589.09	592.70	53	794.53	797.02
4	417.42	421.20	29	590.35	595.07	54	810.10	813.13
5	428.58	430.45	30	600.21	602.45	55	802.23	810.10
6	423.75	427.29	31	590.75	593.30	56	799.22	800.45
7	421.70	428.51	32	593.75	601.06	57	804.23	808.03
8	431.31	439.03	33	596.83	599.70	58	808.17	811.76
9	416.54	423.97	34	587.49	594.74	59	800.98	807.12
10	419.47	425.61	35	597.73	602.56	60	796.38	802.47
11	419.82	423.13	36	591.94	594.45	61	793.33	800.62
12	426.04	429.84	37	592.73	600.94	62	794.22	801.63
13	454.32	449.25	38	632.97	624.98	63	832.44	830.42
14	446.01	442.06	39	629.96	629.15	64	828.35	825.03
15	453.10	449.07	40	632.34	628.31	65	838.35	834.02
16	463.25	460.32	41	629.69	622.83	66	823.49	817.00
17	453.20	450.07	42	622.40	615.98	67	827.16	824.63
18	456.15	454.21	43	628.01	626.77	68	834.32	829.74
19	457.82	453.57	44	624.45	618.78	69	826.32	825.54
20	446.05	445.22	45	623.45	621.66	70	824.79	819.40
21	463.75	457.74	46	629.31	626.29	71	830.05	825.57
22	459.66	456.67	47	621.03	620.33	72	836.49	828.58
23	463.25	456.20	48	629.81	627.24	73	838.89	831.08
24	455.97	449.35	49	626.86	620.30	74	835.17	832.90
25	439.91	440.99	50	605.65	613.27	75	812.77	819.29

Table 4. Mass of the collection bucket for the clean water test with the operating pressure of 100 kPa.

No.	H1-type (g)		No.	H2-type (g)		No.	H3-type (g)	
1	516.36	522.25	26	722.42	729.13	51	957.00	959.24
2	232.49	230.08	27	367.75	374.61	52	952.05	955.84
3	235.88	230.34	28	366.98	365.84	53	952.56	960.82
4	236.63	239.39	29	363.35	359.00	54	969.55	973.34
5	240.23	242.99	30	373.10	366.30	55	963.49	965.69
6	231.18	238.15	31	360.82	352.67	56	959.58	965.10
7	231.05	232.12	32	353.85	351.79	57	952.27	960.45
8	234.68	239.66	33	365.15	360.57	58	961.03	962.90
9	242.17	234.51	34	369.68	371.75	59	952.20	953.94
10	255.22	247.85	35	356.10	364.28	60	958.44	963.44
11	253.42	250.85	36	346.77	352.92	61	970.72	974.71
12	250.74	245.72	37	361.50	364.55	62	968.30	969.63
13	267.17	262.46	38	386.42	378.33	63	991.55	987.93
14	277.23	271.04	39	377.04	371.44	64	995.77	992.07
15	274.47	270.15	40	380.92	377.10	65	994.97	987.56
16	266.52	268.30	41	382.82	385.68	66	977.85	974.98
17	261.13	266.49	42	373.08	378.36	67	986.57	980.37
18	270.35	271.17	43	385.17	392.18	68	987.16	983.89
19	270.24	277.43	44	391.97	393.23	69	993.49	989.90
20	272.12	264.39	45	384.89	380.24	70	989.20	983.00
21	270.66	263.50	46	375.81	373.60	71	998.96	990.63
22	255.70	252.07	47	386.01	384.45	72	988.94	985.09
23	254.32	252.26	48	396.86	394.30	73	977.51	972.98
24	258.23	256.15	49	381.78	383.01	74	982.22	975.78
25	250.67	254.75	50	376.19	369.23	75	971.28	976.78

Table 5. Mass of the collection bucket for the muddy water test when the fertilizer concentration is 0.6 g/L, the sediment content is 1 g/L, and the operating pressure is 40 kPa.

No.	H1-type (g)		No.	H2-type (g)		No.	H3-type (g)	
1	220.36	215.36	26	296.37	299.95	51	500.97	502.83
2	266.27	266.49	27	381.05	383.73	52	499.41	499.85
3	279.66	283.50	28	325.69	330.45	53	507.07	509.17
4	259.07	255.43	29	346.67	346.33	54	504.34	502.34
5	292.48	291.84	30	346.37	346.85	55	508.78	508.38
6	292.00	290.98	31	380.89	385.61	56	502.56	496.60
7	293.52	290.92	32	345.76	354.62	57	494.76	501.62
8	296.95	297.97	33	302.29	307.25	58	491.86	496.26
9	288.06	290.30	34	400.43	407.59	59	493.71	500.31
10	299.93	292.15	35	310.92	306.56	60	499.99	503.13
11	327.44	315.06	36	326.91	325.23	61	494.02	501.78
12	281.37	278.29	37	380.09	376.13	62	489.70	493.74
13	329.61	326.75	38	356.89	351.19	63	483.96	484.26
14	325.77	324.83	39	303.73	308.11	64	493.88	485.34
15	289.66	284.20	40	333.45	337.51	65	490.45	487.29
16	326.38	317.52	41	327.15	335.27	66	490.54	489.88
17	272.83	270.79	42	395.83	387.71	67	502.58	500.92
18	293.91	287.95	43	338.82	333.98	68	500.80	498.56
19	323.65	322.35	44	351.70	342.78	69	492.13	488.45
20	302.43	308.47	45	338.19	331.11	70	498.90	490.30
21	322.61	329.57	46	337.19	329.83	71	485.32	492.60
22	257.28	264.82	47	384.53	382.29	72	493.90	497.32
23	267.31	267.77	48	376.37	377.07	73	500.12	500.86
24	189.99	196.89	49	395.77	392.19	74	487.63	487.97
25	295.52	301.56	50	306.75	300.13	75	491.47	499.59

Table 6. Mass of the collection bucket for the muddy water test when the fertilizer concentration is 0.6 g/L, the sediment content is 2 g/L, and the operating pressure is 100 kPa.

No.	H1-type (g)		No.	H2-type (g)		No.	H3-type (g)	
1	351.41	347.07	26	544.08	544.22	51	693.37	697.81
2	411.31	408.07	27	523.74	522.20	52	650.04	652.96
3	154.57	149.13	28	119.01	116.43	53	685.51	689.19
4	410.14	412.62	29	519.71	518.95	54	613.56	606.90
5	412.64	416.40	30	519.35	522.55	55	644.84	641.82
6	199.66	203.62	31	532.57	538.93	56	681.58	676.28
7	405.64	412.88	32	498.34	506.62	57	681.43	689.67
8	412.88	410.28	33	476.51	485.35	58	637.03	638.23
9	421.16	416.40	34	540.85	540.51	59	627.49	633.59
10	414.20	419.62	35	490.27	488.61	60	664.11	664.97
11	397.71	399.03	36	517.80	515.78	61	690.68	697.50
12	335.81	342.49	37	535.70	528.60	62	684.45	689.97
13	397.96	389.30	38	527.33	520.65	63	680.53	685.53
14	428.60	419.72	39	486.20	492.46	64	700.57	694.97
15	426.80	427.98	40	513.02	519.76	65	636.52	631.94
16	410.63	407.73	41	508.35	510.35	66	694.83	693.97
17	408.67	407.01	42	544.94	542.40	67	662.73	660.15
18	415.29	409.97	43	483.71	479.01	68	660.22	654.56
19	428.72	419.62	44	517.93	516.41	69	671.57	667.49
20	425.03	434.07	45	500.10	498.26	70	684.20	678.98
21	425.29	425.75	46	497.77	492.45	71	682.69	686.13
22	207.89	214.75	47	531.01	527.65	72	660.28	668.20
23	412.33	416.23	48	468.01	468.65	73	647.29	655.67
24	181.86	184.30	49	528.85	530.09	74	648.60	649.86
25	425.91	427.95	50	448.22	453.94	75	656.20	661.94

Table 7. Mass of the collection bucket for the muddy water test when the fertilizer concentration is 0.6 g/L, the sediment content is 3 g/L, and the operating pressure is 70 kPa.

No.	H1-type (g)		No.	H2-type (g)		No.	H3-type (g)	
1	294.60	302.40	26	386.04	383.40	51	492.89	490.63
2	168.37	170.43	27	376.92	381.66	52	496.76	493.34
3	177.33	176.32	28	98.76	101.90	53	567.85	570.47
4	282.01	274.53	29	371.71	370.03	54	537.51	544.45
5	281.16	280.92	30	381.53	387.81	55	557.95	558.39
6	284.55	282.83	31	382.35	376.15	56	559.81	567.31
7	289.71	280.51	32	389.38	387.96	57	540.10	532.56
8	396.08	399.54	33	381.79	378.41	58	553.08	543.86
9	280.13	283.31	34	377.32	380.70	59	440.34	434.14
10	272.27	269.85	35	378.91	378.97	60	546.34	542.94
11	281.01	273.97	36	368.14	373.52	61	462.81	456.53
12	294.13	292.59	37	366.43	370.09	62	228.52	219.84
13	273.66	278.64	38	370.73	374.23	63	526.23	519.53
14	274.40	281.76	39	356.09	349.79	64	505.46	508.88
15	277.80	280.10	40	376.81	369.59	65	517.19	518.43
16	288.42	292.04	41	376.48	376.26	66	508.33	511.29
17	284.92	286.16	42	380.64	387.22	67	549.09	556.15
18	296.01	301.53	43	379.42	372.26	68	545.97	548.39
19	284.54	286.28	44	382.12	384.14	69	411.03	420.29
20	290.29	281.55	45	373.55	382.51	70	546.21	548.69
21	298.31	289.65	46	375.68	383.58	71	541.40	532.64
22	282.31	278.39	47	379.02	388.50	72	560.41	569.89
23	338.40	334.38	48	370.51	361.87	73	548.36	540.64
24	342.35	334.99	49	244.63	236.71	74	549.14	542.28
25	339.58	331.24	50	370.53	366.49	75	590.59	589.21

Table 8. Mass of the collection bucket for the muddy water test when the fertilizer concentration is 1.8 g/L, the sediment content is 1 g/L, and the operating pressure is 100 kPa.

No.	H1-type (g)		No.	H2-type (g)		No.	H3-type (g)	
1	367.25	363.69	26	558.28	559.68	51	731.20	738.80
2	329.10	320.64	27	545.54	537.14	52	749.35	754.55
3	322.20	336.02	28	530.16	522.90	53	797.83	802.51
4	221.42	227.44	29	553.32	546.76	54	736.77	736.27
5	221.39	229.37	30	531.82	537.12	55	744.18	738.74
6	423.56	430.26	31	577.53	577.99	56	725.99	724.89
7	417.53	420.71	32	562.49	555.05	57	726.54	734.14
8	437.67	431.07	33	518.02	527.08	58	615.05	621.05
9	420.91	417.85	34	558.75	553.77	59	674.31	675.19
10	429.13	431.45	35	575.07	570.11	60	707.23	715.33
11	415.61	418.13	36	490.49	488.71	61	689.86	692.58
12	351.61	354.11	37	563.20	557.56	62	708.92	717.84
13	436.56	434.04	38	527.58	522.16	63	715.42	724.26
14	420.26	416.18	39	501.94	513.12	64	718.42	711.56
15	423.05	414.65	40	534.98	540.96	65	717.39	712.01
16	414.66	412.26	41	535.17	543.21	66	742.10	739.64
17	430.69	422.63	42	569.20	561.06	67	713.81	707.27
18	439.65	435.83	43	522.26	515.16	68	712.33	709.37
19	425.85	419.57	44	543.89	542.07	69	653.64	656.86
20	420.97	428.07	45	520.76	518.72	70	688.59	685.99
21	428.40	429.04	46	504.25	501.55	71	721.56	728.18
22	413.74	418.22	47	527.66	523.88	72	734.95	737.03
23	423.63	424.63	48	503.72	507.02	73	669.04	676.58
24	435.14	435.10	49	544.85	553.21	74	743.88	751.46
25	390.12	385.94	50	546.95	548.69	75	773.24	777.30

Table 9. Mass of the collection bucket for the muddy water test when the fertilizer concentration is 1.8 g/L, the sediment content is 2 g/L, and the operating pressure is 70 kPa.

No.	H1-type (g)		No.	H2-type (g)		No.	H3-type (g)	
1	253.36	249.28	26	387.96	392.46	51	266.51	273.49
2	236.86	236.52	27	391.12	382.90	52	614.00	617.82
3	241.18	240.02	28	353.74	353.36	53	273.68	274.44
4	337.42	345.92	29	334.85	329.89	54	618.67	618.45
5	143.54	134.84	30	283.49	290.91	55	615.13	616.51
6	248.26	250.58	31	496.25	497.13	56	610.27	603.09
7	243.17	244.97	32	467.76	469.62	57	575.78	571.46
8	246.35	240.37	33	452.85	457.55	58	584.36	585.44
9	313.25	320.07	34	478.67	473.11	59	483.43	485.23
10	307.50	308.82	35	445.59	439.37	60	594.60	595.58
11	340.16	341.96	36	463.57	460.51	61	537.91	538.71
12	342.14	348.22	37	385.66	383.70	62	597.40	603.60
13	250.92	242.38	38	377.97	370.43	63	584.41	588.19
14	144.80	149.26	39	429.40	435.04	64	584.60	578.40
15	224.93	216.35	40	461.76	467.36	65	580.57	571.77
16	358.06	357.14	41	424.59	429.25	66	255.38	248.94
17	316.66	313.16	42	453.83	449.39	67	487.89	483.63
18	331.54	326.10	43	439.31	437.31	68	242.50	241.16
19	329.83	327.71	44	441.16	436.50	69	279.95	271.47
20	326.38	334.52	45	499.93	498.25	70	585.79	580.77
21	319.18	323.76	46	403.25	395.51	71	565.58	574.42
22	323.81	327.83	47	486.24	481.94	72	570.92	573.52
23	332.11	336.31	48	455.26	461.24	73	588.38	591.08
24	318.60	323.30	49	484.25	485.49	74	585.40	586.60
25	321.41	325.31	50	465.39	470.53	75	607.60	616.14

Table 10. Mass of the collection bucket for the muddy water test when the fertilizer concentration is 1.8 g/L, the sediment content is 3 g/L, and the operating pressure is 40 kPa.

No.	H1-type (g)		No.	H2-type (g)		No.	H3-type (g)	
1	242.09	241.53	26	309.10	314.30	51	424.14	427.92
2	243.96	241.60	27	276.23	268.77	52	303.93	301.27
3	170.67	162.25	28	255.52	251.94	53	415.11	420.69
4	166.66	168.38	29	145.27	148.87	54	172.62	168.12
5	168.25	166.79	30	142.91	151.57	55	162.03	165.25
6	239.93	246.11	31	86.43	94.73	56	163.47	154.97
7	253.27	258.27	32	289.41	286.19	57	433.62	437.36
8	198.62	197.62	33	337.73	342.35	58	416.64	418.26
9	128.70	133.66	34	350.22	351.26	59	431.26	437.12
10	227.34	226.08	35	287.73	283.73	60	447.51	455.65
11	267.37	271.01	36	331.01	327.59	61	465.31	468.67
12	266.10	269.72	37	323.54	323.02	62	145.82	146.92
13	116.56	116.08	38	304.35	303.37	63	326.56	330.32
14	257.73	257.19	39	294.10	295.34	64	153.03	150.21
15	125.87	127.57	40	139.26	143.82	65	452.09	443.69
16	274.97	273.39	41	149.89	150.33	66	444.71	443.69
17	249.88	254.78	42	375.73	370.11	67	466.11	461.53
18	271.67	273.99	43	141.34	132.68	68	404.88	403.82
19	256.36	251.12	44	317.05	316.65	69	420.98	417.74
20	255.60	261.04	45	315.41	311.29	70	419.59	416.45
21	253.34	254.70	46	334.73	326.51	71	429.33	427.99
22	253.00	254.02	47	347.89	343.61	72	450.78	453.90
23	266.89	268.95	48	307.17	311.73	73	448.17	453.37
24	162.25	169.53	49	398.65	397.25	74	484.30	485.92
25	147.58	144.26	50	264.68	270.08	75	441.93	440.67

Table 11. Mass of the collection bucket for the muddy water test when the fertilizer concentration is 3 g/L, the sediment content is 1 g/L, and the operating pressure is 70 kPa.

No.	H1-type (g)		No.	H2-type (g)		No.	H3-type (g)	
1	291.69	289.43	26	467.70	461.38	51	415.01	416.89
2	296.73	305.29	27	434.18	428.34	52	223.08	227.70
3	290.98	285.92	28	421.39	417.89	53	549.68	557.44
4	281.85	286.53	29	431.09	424.59	54	584.78	579.26
5	291.40	292.68	30	442.99	449.71	55	592.14	590.10
6	293.17	299.23	31	449.07	452.31	56	549.63	544.61
7	295.58	302.18	32	422.86	426.44	57	550.91	551.21
8	301.85	298.13	33	387.15	385.55	58	556.04	560.20
9	295.94	287.72	34	362.59	354.85	59	561.26	568.66
10	294.35	296.49	35	374.92	372.46	60	564.38	568.12
11	296.89	302.77	36	429.10	422.02	61	530.58	533.72
12	301.09	307.51	37	363.06	360.36	62	559.46	565.54
13	298.19	296.35	38	404.26	403.38	63	571.19	574.65
14	307.16	306.22	39	391.24	395.68	64	561.07	553.83
15	308.73	303.55	40	421.73	423.47	65	553.60	550.72
16	303.84	303.48	41	413.20	421.94	66	551.61	547.01
17	304.96	304.36	42	452.64	446.12	67	552.78	547.62
18	302.81	298.15	43	456.15	454.17	68	538.46	533.38
19	289.11	290.91	44	437.50	433.60	69	549.96	543.88
20	301.30	301.62	45	424.51	422.41	70	555.48	551.38
21	300.59	300.57	46	423.54	421.18	71	559.96	566.88
22	306.51	315.59	47	451.05	445.07	72	560.04	564.50
23	315.12	318.10	48	392.41	394.21	73	565.05	568.97
24	311.18	314.48	49	483.21	488.29	74	582.33	586.77
25	304.19	313.11	50	499.19	508.19	75	542.96	549.14

Table 12. Mass of the collection bucket for the muddy water test when the fertilizer concentration is 3 g/L, the sediment content is 2 g/L, and the operating pressure is 40 kPa.

No.	H1-type (g)		No.	H2-type (g)		No.	H3-type (g)	
1	228.20	224.58	26	320.72	318.82	51	211.14	218.08
2	221.30	220.20	27	214.90	217.34	52	208.64	216.78
3	225.60	220.14	28	284.50	278.04	53	199.73	209.79
4	228.75	227.79	29	187.60	183.34	54	457.51	455.35
5	227.56	235.26	30	353.08	357.86	55	398.49	396.21
6	225.07	231.07	31	209.70	215.26	56	511.87	510.35
7	228.00	231.82	32	351.53	356.33	57	439.53	439.95
8	199.55	196.21	33	284.85	286.13	58	336.24	337.62
9	208.14	204.56	34	284.22	289.48	59	451.71	457.71
10	239.99	247.75	35	343.09	334.81	60	433.76	436.52
11	118.27	111.07	36	362.21	354.47	61	462.83	471.13
12	115.77	110.67	37	280.25	280.29	62	219.20	215.50
13	210.51	208.63	38	358.12	350.46	63	428.96	437.20
14	245.16	241.16	39	314.50	323.08	64	361.42	359.64
15	267.48	261.38	40	255.08	257.76	65	407.96	402.52
16	179.65	170.17	41	208.51	209.23	66	427.60	422.22
17	184.30	177.60	42	282.34	280.34	67	286.75	281.99
18	226.29	218.95	43	339.73	335.29	68	286.11	287.15
19	269.04	262.16	44	352.88	344.14	69	420.73	411.77
20	271.53	273.35	45	348.73	343.89	70	438.85	438.13
21	274.20	277.30	46	270.20	262.16	71	431.07	436.29
22	281.25	286.55	47	363.02	361.78	72	434.53	441.93
23	275.83	282.11	48	340.25	343.89	73	446.76	440.76
24	271.20	278.44	49	294.60	292.36	74	433.48	435.44
25	230.17	243.85	50	289.68	294.56	75	369.72	365.98

Table 13. Mass of the collection bucket for the muddy water test when the fertilizer concentration is 3 g/L, the sediment content is 3 g/L, and the operating pressure is 100 kPa.

No.	H1-type (g)		No.	H2-type (g)		No.	H3-type (g)	
1	313.87	319.73	26	425.95	422.23	51	571.90	567.28
2	101.68	107.72	27	400.61	398.75	52	131.13	135.71
3	318.14	310.72	28	111.96	114.90	53	542.19	546.81
4	97.01	97.17	29	403.26	405.90	54	561.14	553.74
5	303.74	304.88	30	401.44	405.82	55	501.95	501.21
6	280.82	287.54	31	410.77	413.09	56	507.86	504.42
7	316.89	324.65	32	406.54	412.48	57	594.44	593.60
8	262.51	254.21	33	326.25	327.29	58	569.90	573.00
9	314.25	312.41	34	430.92	429.24	59	570.22	570.84
10	315.19	324.09	35	365.43	363.23	60	604.41	608.09
11	317.36	322.56	36	380.56	375.50	61	623.44	624.50
12	327.24	329.68	37	430.06	427.92	62	586.72	592.24
13	302.46	298.44	38	404.09	396.99	63	574.76	576.64
14	309.29	302.33	39	386.58	392.28	64	134.72	128.18
15	95.15	98.97	40	413.44	417.92	65	577.72	569.28
16	105.46	103.72	41	391.30	400.28	66	131.69	131.09
17	324.93	319.99	42	443.60	438.46	67	136.63	127.81
18	292.87	291.73	43	396.27	389.73	68	235.81	234.41
19	306.93	302.99	44	382.10	381.68	69	571.96	569.94
20	320.86	323.72	45	389.34	385.74	70	581.26	573.58
21	324.88	329.84	46	368.79	365.35	71	595.05	597.67
22	306.64	307.46	47	403.97	398.69	72	592.16	597.16
23	312.47	316.53	48	340.75	348.41	73	565.97	568.13
24	310.59	317.57	49	409.94	418.94	74	561.70	569.52
25	295.95	297.39	50	359.29	367.11	75	552.72	557.46
